# Nutritional enhancement of leaves by a psyllid through senescence-like processes: insect manipulation or plant defence?

**DOI:** 10.1007/s00442-014-3087-3

**Published:** 2014-09-21

**Authors:** M. J. Steinbauer, A. E. Burns, A. Hall, M. Riegler, G. S. Taylor

**Affiliations:** 1 Department of Zoology, La Trobe University, Melbourne, Vic 3084 Australia; 2 Hawkesbury Institute for the Environment, University of Western Sydney, Penrith, NSW 2751 Australia; 3 Australian Centre for Evolutionary Biology and Biodiversity, The University of Adelaide, Adelaide, SA 5005 Australia

**Keywords:** Amino nitrogen, Chlorosis, Defoliation, Photosynthetic pigments, Insect performance

## Abstract

Some herbivores can modify the physiology of plant modules to meet their nutritional requirements. Induction of premature leaf senescence could benefit herbivores since it is associated with the mobilisation of nutrients. We compared the effects of nymphal feeding by *Cardiaspina* near *densitexta* on *Eucalyptus moluccana* with endogenous processes associated with senescence to assess the relative merits of an insect manipulation or plant defence interpretation of responses. Evidence supporting insect manipulation included increased size of fourth and fifth instar nymphs (in the latter the effect was restricted to forewing pad length of females) on leaves supporting high numbers of conspecifics and feeding preventing leaf necrosis. Intra-specific competition negated greater performance at very high densities. High and very high abundances of nymphs were associated with increased concentrations of amino acid N but only very high abundances of nymphs tended to be associated with increased concentrations of six essential amino acids. Contrary to the insect manipulation interpretation, feeding by very high abundances of nymphs was associated with significant reductions in chlorophyll, carotenoids and anthocyanins. Evidence supporting plant defence included the severity of chlorosis increasing with the abundance of nymphs. Leaf reddening did not develop because ambient conditions associated with photoinhibition (high irradiance and low temperature) were not experienced by leaves with chlorotic lesions. Leaf reddening (from anthocyanins) alone is not expected to adversely affect nymphal survival; only leaf necrosis would kill nymphs. For senescence-inducing psyllids, nutritional enhancement does not fit neatly into either an insect manipulation or plant defence interpretation.

## Introduction

The physiology of foliage determines its nutritional quality to herbivores. It is for this reason that many herbivores exhibit specific preferences for the age of the plant modules they eat. Some herbivores have been found to select leaves on the basis of their nutritional quality rather than on the basis of their age. Kennedy and Booth (1951) and Kennedy (1958) found that, on both their summer and winter host plants, *Aphis fabae* Scop. preferred to feed and reproduced faster on young as well as early senescent leaves compared to mature leaves; in both instances the availability of amino acid N was central to host utilisation. Nevertheless, amino acid availability cannot entirely explain the utilization of senescing hosts; changes in concentrations of secondary metabolites such as phenolic compounds are also important (Zucker 1982; Taylor 1997; Sandström 2000).

Leaf senescence is the final stage of leaf development; it is a controlled process characterised by numerous interdependent structural and biochemical changes, the onset of which can be initiated by external stressors, including wounding (Lim et al. 2007; Thomas et al. 2009). The visible symptom of senescence is leaf yellowing which is linked to declining chlorophyll content caused by the death of chloroplasts in mesophyll cells (Guo and Gan 2005; Lim et al. 2007). The yellow-orange colouration of senescing leaves, which is unmasked by chlorophyll catabolism, is due to carotenoid pigments found in plastids. The red colouration of the senescing leaves of deciduous plant species is caused by the de novo synthesis of anthocyanins in cell vacuoles (Archetti et al. 2009). The remainder of the symptoms of leaf senescence are invisible to the human observer—these include protein and lipid degradation and the export of sucrose. For herbivores, it is the rise in concentrations of soluble proteins that increase the nutritional quality of senescing leaves. However, only insects that are able to perceive changes in leaf colour and which are able to reach senescing leaves quickly may benefit from higher concentrations of nutrients (Holopainen et al. 2009). The mobilisation of N in evergreen plants such as eucalypts can occur independent of leaf senescence, without a decrease in chlorophyll or Rubisco concentrations and no reduction in photosynthetic capacity (Wendler et al. 1995; Crawford and Wilkens 1996).

The capacity to induce premature leaf senescence could be a beneficial adaptation to acquire essential nutrients. Unlike ‘senescence-feeders’ (White 2009), e.g. *A. fabae* (Kennedy and Booth 1951; Kennedy 1958), senescence-inducing herbivores do not need to locate mature leaves affected by environmental stressors. Feeding by many species of Hemiptera, especially by those belonging to the suborder Sternorrhyncha, is often associated with yellowing and premature leaf senescence (Jiménez et al. 1995; Jones et al. 2000; Ni et al. 2001; Liu et al. 2006; Nissinen et al. 2007). In Australia, *Cardiaspina* psyllid nymphs are renowned for their capacity to cause leaf discolouration and premature abscission resulting in serious defoliation of eucalypts (Taylor 1962; Morgan and Taylor 1988; Marsh and Adams 1995). Using light microscopy, Woodburn and Lewis (1973) found that feeding by *Cardiaspina* nymphs was associated with the breakdown of palisade mesophyll and that this damage resembled that observed in naturally senescing leaves; they postulated that *Cardiaspina* species may have different nutritional requirements to psyllid species which do not cause apparent phytotoxicosis, e.g. *Glycaspis* species. Using transmission electron microscopy, Crawford and Wilkens (1996) found that psyllid-induced leaf senescence exhibited important differences in cellular ultrastructure compared to natural leaf senescence. Chloroplasts remained intact and apparently functional in naturally senescing leaves whereas lysis of thylakoid membranes in chloroplasts was observed in mesophyll cells affected by psyllid salivary enzymes. Feeding by *Cardiaspina* nymphs is considered most akin to ‘lacerate and flush’ feeding (Gary Taylor, personal communication; Miles and Taylor 1994). That is, stylets exit from a short stylet sheath, pierce nearby cells and the contents are mixed with watery saliva and sucked back. Larson and Whitham (1991) noted, when comparing free-living and gall-forming (sessile) aphids, that the sessile biology of *Cardiaspina* nymphs could hinder their survival and development if host modules were inherently poor quality and nymphs themselves were not able to enhance the nutritional quality of their ingesta. Leaf reddening may follow in the same locations where the chlorotic lesions caused by feeding nymphs develop (White 1970; Morgan and Taylor 1988; Crawford and Wilkens 1996). In the case of one *Glycaspis* species, leaf reddening has been observed to be more noticeable in late winter (Moore 1961) while Morgan (1984) reported that the reddish lesions of *Cardiaspina* species could be prevented from becoming necrotic through the provision of N fertilizer. The accumulation of anthocyanins in response to cold stress and nutrient deficiency reflects their role in photoprotection (Close and Beadle 2003). The red colouration of insect-induced galls has been suggested to reflect a down-regulation of photosynthesis associated with the degradation of chlorophyll (Connor et al. 2012). In this instance, the galling insect is thought to produce exogenous cytokinins which initiate the up-regulation of anthocyanin synthesis.

Yellowing and premature leaf senescence are most commonly interpreted as evidence of host wounding, possibly of nutritional benefit to the herbivore (White 1970; Woodburn and Lewis 1973; Crawford and Wilkens 1996; Taylor 1997; Sandström et al. 2000; Nissinen et al. 2007). Nevertheless, another viewpoint has also been posited, specifically that the export of nutrients from senescing leaves could be an adaptation to limit aphid growth (Pegadaraju et al. 2005). If psyllids manipulate the nutritional quality of the plant, then the following hypotheses might be posited: no chlorosis leading to reduced photosynthesis will be evident (H1), free amino acids (FAAs) required by psyllids should increase in concentration relative to those less important to their survival and development (H2), and psyllids should benefit from the modifications to leaf quality that they induce (H3). If, however, plant responses to psyllid feeding were primarily defensive in nature, the following alternate hypothesis might be posited: the intensity of the plant’s response will be related to the intensity of feeding damage (determined either by the number of psyllids per unit area of leaf surface and by their per capita ability to injure the host) (H4). We sought evidence supporting either an insect manipulation or plant defence interpretation of psyllid-induced leaf senescence.

## Materials and methods

### Field sites

Natural stands of *Eucalyptus moluccana* Roxb. (grey box; subgenus *Symphyomyrtus*, section *Adnataria*) at three sites in Western Sydney’s Cumberland Plain Woodland, New South Wales, were chosen for this study; the sites included Mount Annan Botanical Gardens (MAB; 150°46′8.46″E, 34°3′45.60″S), Bligh Park (BP; 150°47′26.30″E, 33°37′59.49″S) and Gleesons Trees Reserve (GTR; 150°54′15.77″E, 33°47′29.05″S). The Cumberland Plain Woodland of which *E. moluccana* is a characteristic species occurs on silty-clayey sands and gravel overlaying the Wianamatta group shales; annual rainfall varies from >800 to approximately 900 mm and average temperature maxima in January are between 27.6 and 29.5 °C (Tozer 2003). At each site, six sapling (understorey) trees between 10- and 50-m distance from each other were selected for study; all were comparably shaded by surrounding mature trees. While the study psyllid species was the most abundant insect herbivore, saplings at all sites were occupied by a range of other insect taxa but none were in high abundance or causing substantial defoliation. Each site differed in psyllid abundance from almost none (MAB), to high (BP) to very high numbers (GTR). Sites were visited during the Austral spring, i.e. 15–16 October and 18–20 November 2012.

### Leaf selection and psyllid surveys

Six pairs of leaves, at nodes four to six distant from the apical bud, on separate branches were tagged on each sapling. By choosing leaf pairs, we minimised age-related differences in nutritional quality and susceptibility to psyllid feeding. All leaves at GTR supported at least one live nymph in October 2012 while 33/36 leaves at BP supported at least one live nymph in October 2012. The leaf furthest from the end of each branch (slightly older of the pair) was harvested in October and the other (slightly younger of the pair) was harvested approximately 1 month later. Nitrile examination gloves were worn when harvesting leaves and each was stored in a zip-lock plastic bag and kept on dry ice prior to return to the laboratory.

The lerps (small shelters constructed by nymphs using carbohydrates secreted from the anus) of the *Cardiaspina* species we studied most closely resembled those of *C. densitexta* Taylor (Hemiptera: Aphalaridae) which was originally described from *Eucalyptus fasciculosa* F. Muell. from south-eastern Australia. Live nymphs and empty lerps on each leaf half were counted and recorded separately. When nymphs and lerps had been enumerated, they were removed from leaves using forceps and preserved in 70 % ethanol for instar determination and morphometric measurement.

### Foliar pigments and nutritional analyses

After nymphs were removed from leaves, lamellae were cut from the midrib and one leaf half placed in a labelled plastic bag at −20 °C (for foliar pigment analysis) and the other half was stored in a labelled envelope, placed in a microwave on high (1,100 W) for 30 s before being oven dried at 70 °C for 24 h (for amine analysis).

Frozen leaf halves were analysed for total chlorophyll, carotenoids and anthocyanins. For leaves collected in October, anthocyanins were extracted from the tip and distal portion of each leaf while chlorophylls and carotenoids were extracted from the remainder (all sites). For leaves collected in November, the most chlorotic and reddish portions of leaves were used for pigment extractions (BP and GTR only; chlorosis and reddening were not evident on leaves from MAB). Chlorophylls and carotenoids were extracted simultaneously by grinding 100 mg of leaf with a small volume of liquid N and 1.0 mL of 80 % acetone (pH 7.8, adjusted with 3.2 % hydrochloric acid and 1 M potassium hydroxide and buffered with 2.5 mM potassium orthophosphate) using a mortar and pestle. 2.0 mL of homogenate was transferred to a test tube, vortexed for 30 s and centrifuged for 10 min at 2,500 r.p.m. The supernatant was removed and stored in 5 mL plastic vials at −20 °C until analysis. Absorbance by chlorophylls *a* and *b* was measured at 646.6 and 663.6 nm, respectively, using a spectrophotometer. Chlorophyll concentrations were calculated using formulae given by Porra et al. (1989). Absorbance by carotenoids was measured at 470 nm and concentration calculated after Lichenthaler (1987). Anthocyanins were also extracted by grinding as described above but with 1.0 mL of 100 % acidified ethanol (pH 1.0, adjusted with 3.2 % hydrochloric acid). Leaf homogenate was immersed in boiling water for 1.5 min and kept in the dark for 24 h at approximately 4 °C to extract. Samples were centrifuged and stored as described above prior to analysis. Absorbance by anthocyanins was measured at 535 nm using a spectrophotometer. A calibration curve using kuromanin chloride [cyanidin-3-glucoside; which occurs in several *Eucalyptus* species (Sharma and Crowden 1974)] was used to calculate anthocyanin concentrations. To convert concentrations of foliar pigments to micrograms per milligram of leaf tissue (μg mL^-1^), concentrations were multiplied by the dilution factor and the volume of solvent (mL) and divided by the weight of leaf material (mg).

The nutritional quality of leaves was assessed by measuring concentrations of FAAs. Concentrations of 19 of the 20 common amino acids and three other amine-group-containing metabolites [the latter comprising 4-hydroxy-proline, ornithine and γ-aminobutyric acid (GABA)] were quantified following the technique described by Steinbauer (2013). Responses for cysteine were poor and often below limits of detection or quantitation. The amino acids designated herein as essential are those identified by Douglas (2006). We summarise our data according to their possible significance to psyllid nymphs (i.e. concentrations of all amino acids plus amine metabolites as well as the percentage essential amino acids) and as indicators of leaf senescence or stress (i.e. concentrations of asparagine (Asn) plus glutamine (Gln) and proline (Pro). According to Guo and Gan (2005), the major amino acid involved in the transport of N from senescing leaves is Gln and, to a lesser extent, Asn. Concentrations of both these amino acids increased in aphid-infested bean plants (Leroy et al. 2011). Increased concentrations of Pro have been reported in plant modules fed upon by aphids and psyllids and have been used as an indicator of herbivore stress (Cabrera et al. 1995; Yang et al. 2011). Pro concentrations also increase in plants experiencing drought stress (Miles et al. 1982; Cabrera et al. 1995). Where evident on leaves collected in November, the most chlorotic and reddish portions of leaves were used for FAA analysis (BP and GTR only).

### Nymphal morphometrics

Nymphs were identified to instar (I–V) according to the development of their wing pads. We measured head width (HW), forewing pad length (FWL; poorly developed in first and second instar nymphs) and body length (BL) of nymphs to infer the effect of the nutritional quality of hosts on performance. Morphometric measurements (mm) were made using a Leica MZ16 dissecting microscope at 115× magnification.

### Statistical analyses

Abundances of nymphs were square root (count +0.5) transformed prior to statistical analysis. Due to some missing count data, the abundance of nymphs at BP and GTR was compared using general linear models (GLMs) with month and tree (nested within month) as factors (Minitab 17.1.0). Month was treated as a random factor while Tree was treated as fixed factor. We also compared abundances per tree using repeated-measures GLMs followed by Tukey B post hoc tests (SPSS 21.0.0.0).

Although leaves harvested in October and November belonged to a pair arising from the same node, concentrations of foliar pigments and FAAs were not treated as paired observations because psyllid abundance differed significantly between leaves (as well as between halves of the same leaf). Concentrations of pigments (all untransformed) were first compared using GLMs with Month and Tree (nested within Month) as factors. Per tree concentrations of pigments were also analysed using repeated-measures GLMs followed by Tukey B post hoc tests. The same analyses were applied to the FAA and amines data. Concentrations were log_10_ (concentration +1) transformed while percentages were arcsine transformed prior to analysis.

The gender of fifth instar nymphs could not be reliably determined based on their morphology. We assumed male nymphs would be smaller than female nymphs because the BL (head to tip of folded wings) of adult males is less (ca. 2.6 mm) than that of adult females (ca. 3.4 mm) (Taylor 1962). For fifth instar nymphs from each site, we used principal components analysis with a covariance matrix to identify groupings of individuals (Minitab 17.1.0). The first two components explained 99.2 % of the total variance in measurements for fifth instar nymphs from BP while those for nymphs from GTR explained 98.4 % of the total variance. The second component score from each principal components analysis was used to identify morphometric data for probable males (smaller) and females (larger) within the original data set. Morphometric data for each nymphal instar were unbalanced. Differences in the size of morphological features for the same instar and, within fifth instars, genders from BP or GTR were compared using general multivariate ANOVA (MANOVA) followed by one-way ANOVA (Minitab 17.1.0). Anderson–Darling normality tests were used to examine the distributions of morphometric measurements. Pillai’s trace test statistic is reported for each MANOVA result. There were too few (*n* = 5) third instar nymphs from BP for measurement so comparisons of nymphs of this instar from BP and GTR could not be made.

Since the concentrations of many FAAs are inter-correlated, factor analysis using the principal extraction technique based on covariance matrices and varimax rotation was performed before considering possible explanatory relationships (SPSS 21.0.0.0). Data for all three field sites and both harvest dates were included in this analysis so changes in FAA composition in the presence (BP and GTR) or absence (MAB) of feeding psyllids could be identified (see Sandström et al. 2000). Stepwise regressions using component factor scores were subsequently used to consider relationships between the abundances of nymphs and groupings of FAAs. The November data for counts of remaining live nymphs and abandoned lerps were combined to reflect the total feeding activity leaves had experienced. This analysis was repeated for the three foliar pigments but only one component (total chlorophyll) was extracted. Component factor scores for total chlorophyll were regressed against transformed nymphal abundances using simple linear regression.

## Results

### Development and abundance of nymphs

In October, all nymphs at BP were at either first or second instar. By November, second through to fifth instar nymphs were present at BP. At GTR in October, nymphs at first through to fourth instar were present. Fifth instar nymphs were present at GTR by November. Many nymphs at both sites had abandoned their lerps and eclosed by November. Only small numbers of early instar nymphs were found at MAB in October and November, i.e. there were first instar nymphs on 3/36 leaves in October and first to second instar nymphs on 10/36 leaves in November (data not analysed).

**Fig. 1:**
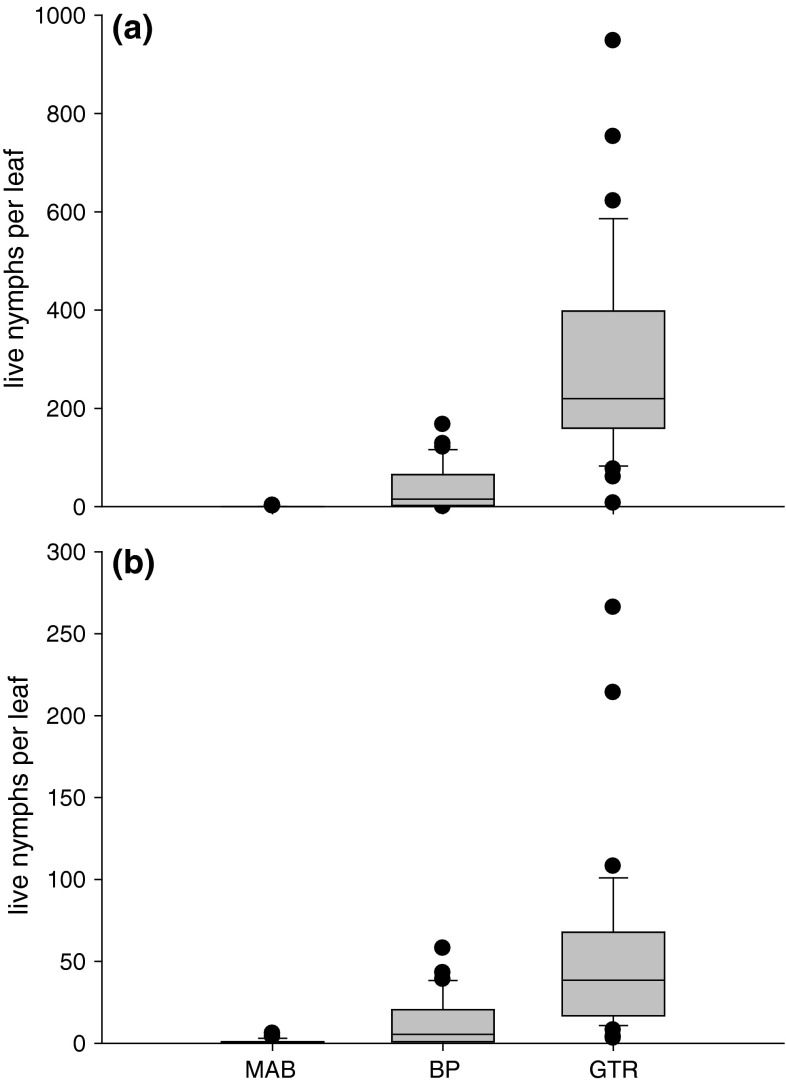
Abundances (±SE) of psyllid nymphs on *Eucalyptus moluccana* leaves at three sites in **a** October 2012 and **b** November 2012. Results of statistical analyses are given in Table 1.* MAB* Mount Annan Botanical Gardens,* BP* Bligh Park,* GTR* Gleesons Trees Reserve

Abundance at BP varied from one to 167 live nymphs per leaf in October and from one to 58 live nymphs per leaf in November (Fig. 1a, b). Abundance at GTR varied from seven to 948 live nymphs per leaf in October and from three to 266 live nymphs per leaf in November (Fig. 1a, b). GLM analyses indicated that differences in the abundance of nymphs at BP between months and trees were statistically non-significant while differences in abundance at GTR were statistically significant (Table 1). Repeated-measures GLMs indicated that differences in abundance between trees at BP and GTR were statistically non-significant and significant, respectively (Table 1). Leaves from BP each supported an average of 23.9 nymphs. Leaves from tree 2 at GTR supported an average of 380.1 nymphs whereas leaves of tree 6 supported an average of 61.3 nymphs.

**Table 1:** Abundance of *Cardiaspina* near *densitexta* nymphs at Bligh Park (*BP*) (high abundance) and Gleesons Trees Reserve (*GTR*) (very high abundance)

	*df*	*F*	*P*	Homogeneous subsets of trees^a^		
				A	B	C
GLM results						
BP: Month	1, 59	4.39	0.062^b^			
BP: Tree (Month)	10, 59	1.79	0.083			
GTR: Month	1, 59	15.51	0.003^b^			
GTR: Tree (Month)	10, 59	12.25	<0.001			
Repeated-measures GLM results						
BP: Tree	5, 29	2.43	0.059	All trees (23.9)		
GTR: Tree	5, 29	14.66	<0.001	6 (61.3)	4, 3, 1, 5	2 (380.1)

*GLM *General linear model

^a^ *Numbers within a row* identify individual trees at each site;* numbers in parentheses* are per tree average psyllid abundances

^b^Not an exact *F*-test (due to missing values)

### Changes in foliar pigments

**Fig. 2:**
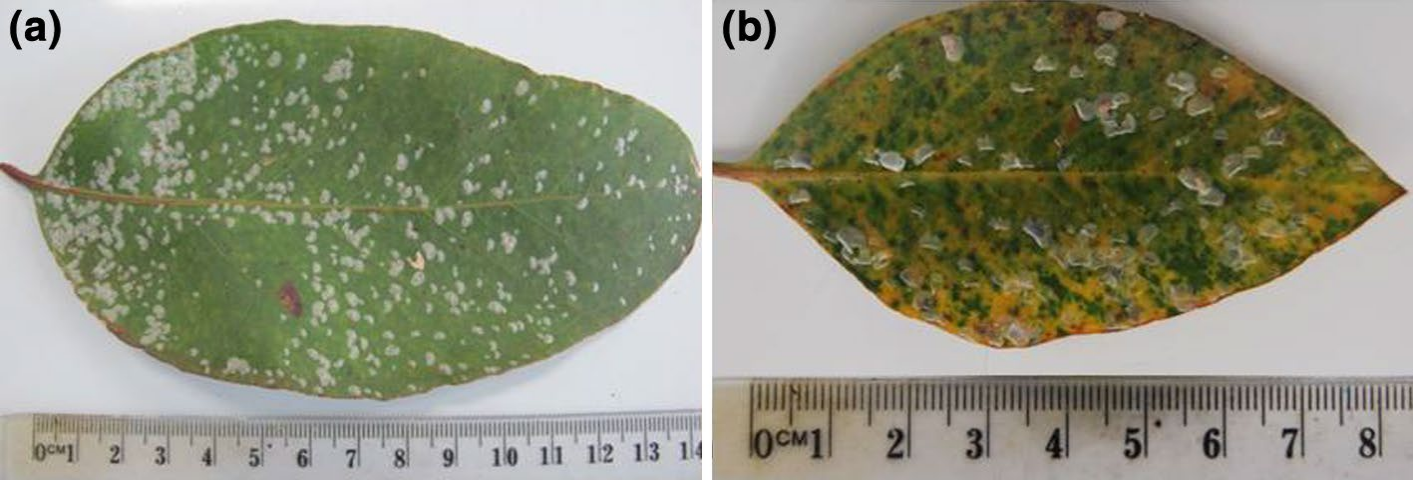
Images of **a** psyllid-infested leaf, October 2012, with chlorotic lesions just visible (all lerps occupied by nymphs) and **b** psyllid-infested leaf, November 2012, with chlorotic lesions clearly visible (only four lerps still occupied by nymphs, remainder abandoned). Note: the* red* (anthocyanic) lesion in the bottom half of (**a**) is associated with an abandoned lerp (of a nymph eclosed earlier in the season) on the abaxial side of the leaf

In October, chlorotic lesions beneath lerps on leaves at BP and GTR were faintly visible to the naked eye (Fig. 2a). By November, 16.7 % of leaves at BP and 94.4 % of leaves at GTR exhibited obvious chlorotic, often coalesced, lesions associated with lerps (Fig. 2b). Little reddening and no necrosis of leaves was evident by November.

**Fig. 3:**
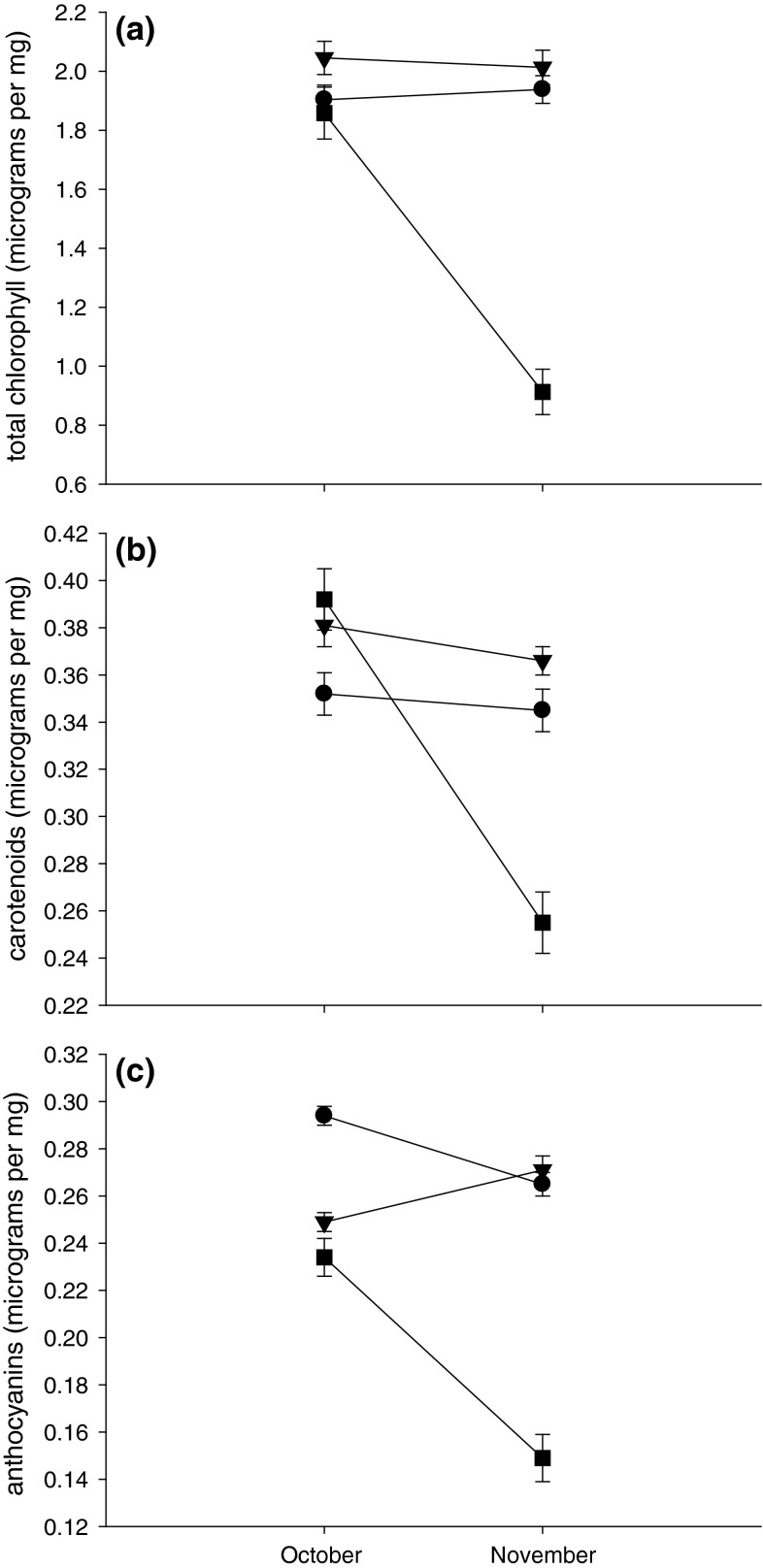
Concentrations (±SE) of **a** total chlorophyll, **b** carotenoids and **c** anthocyanins in leaves of *E. moluccana* at three sites in October and November 2012 (*circles* for leaves from MAB, *triangles* for leaves from BP and *squares* for leaves from GTR). Results of statistical analyses given in Table 2. For abbreviations, see Fig. 1

Where there were almost no psyllids (MAB), neither chlorophyll nor carotenoid concentrations decreased significantly between October and November (Fig. 3a, b; Table 2). However, anthocyanin concentrations at MAB decreased between the two harvests (Fig. 3c). Concentrations of chlorophyll and carotenoids at BP, where there were high numbers of nymphs per leaf, exhibited comparable trends to those at MAB and did not differ significantly between months (Fig. 3a, b; Table 2). Concentrations of anthocyanins at BP did not change significantly between October and November (Fig. 3c). At GTR, concentrations of all three foliar pigments decreased significantly between October and November (Fig. 3a–c; Table 2).

**Table 2:** Concentrations of foliar pigments in *Eucalyptus moluccana* at MAB (almost no psyllids), BP (high abundance) and GTR (very high abundance)

	*df*	*F*	*P*	Homogeneous subsets of trees^a^		
				A	B	C
GLM results						
Total chlorophyll						
MAB: Month	1, 60	0.09	0.775			
MAB: Tree (Month)	10, 60	4.44	<0.001			
BP: Month	1, 59	0.04	0.844^b^			
BP: Tree (Month)	10, 59	3.76	0.001			
GTR: Month	1, 59	12.90	0.005^b^			
GTR: Tree (Month)	10, 59	15.87	<0.001			
Carotenoids						
MAB: Month	1, 60	0.10	0.763			
MAB: Tree (Month)	10, 60	4.00	<0.001			
BP: Month	1, 59	1.24	0.291^b^			
BP: Tree (Month)	10, 59	1.86	0.069			
GTR: Month	1, 59	12.38	0.006^b^			
GTR: Tree (Month)	10, 59	10.49	<0.001			
Anthocyanins						
MAB: Month	1, 60	5.34	0.043			
MAB: Tree (Month)	10, 60	5.50	<0.001			
BP: Month	1, 59	4.32	0.064			
BP: Tree (Month)	10, 60	2.68	0.009			
GTR: Month	1, 58	10.49	0.009^b^			
GTR: Tree (Month)	10, 58	8.04	<0.001			
Repeated-measures GLM results						
Total chlorophyll						
MAB: Tree	5, 30	5.60	0.001	3 (1.62)	6, 4, 2, 1	5 (2.23)
BP: Tree	5, 29	5.59	0.001	5 (1.78)	1, 4	6, 3, 2 (2.33)
GTR: Tree	5, 29	32.58	<0.001	2 (0.88)	5, 3, 1, 4	6 (2.14)
Carotenoids						
MAB: Tree	5, 30	5.33	0.001	3 (0.30), 6	4, 2, 1, 5 (0.41)	
BP: Tree	5, 29	2.97	0.028	4 (0.34)	1, 5, 6, 3, 2 (0.41)	
GTR: Tree	5, 29	22.21	<0.001	2 (0.24)	5, 3, 4, 1	6 (0.44)
Anthocyanins						
MAB: Tree	5, 30	6.66	<0.001	3 (0.26), 1	2, 6, 5, 4 (0.31)	
BP: Tree	5, 30	2.31	0.069	All trees (0.26)		
GTR: Tree	5, 28	10.15	<0.001	2 (0.15), 5, 3, 1, 4	6 (0.26)	

For abbreviations, see Table 1

^a^ *Numbers within a row* identify individual trees at each site;* numbers in parentheses* are per tree average concentrations (µg mg^−1^)

^b^Not an exact *F*-test (due to missing values)

GLMs and repeated-measures GLMs indicated that, in the main, between-tree differences in foliar pigments were statistically significant (Table 2). In the virtual absence of psyllids (MAB), some trees (e.g. tree 3) had consistently lower concentrations of pigments than others (trees 5 or 4). Where there were high and comparable numbers of nymphs per leaf (BP, average of 23.9 nymphs), the ranking of a tree relative to other trees was not consistent indicating that fluctuations in foliar pigments exhibited unique responses to the level of psyllid infestation. In contrast, at GTR, the ranking of trees was consistent across all three foliar pigments with their ranking reflecting their level of infestation and leaf damage. For example, tree 2 had the lowest total chlorophyll, carotenoids and anthocyanins concentrations as well as the highest average nymphal abundance per leaf whereas tree 6 had the highest total chlorophyll, carotenoids and anthocyanins concentrations as well as the lowest average nymphal abundance per leaf.

### Changes in FAAs and amines

**Fig. 4:**
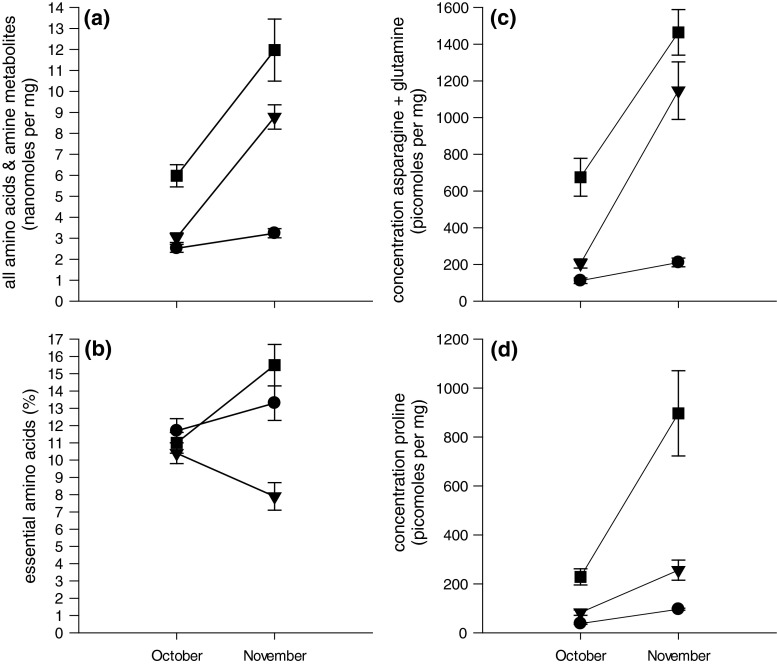
Concentrations (±SE) of **a** all free amino acids (FAAs) and amine metabolites, **b** proportional composition of essential FAAs, **c** asparagine and glutamine and **d** proline in leaves of *E. moluccana* at three sites in October and November 2012 (*circles* for leaves from MAB, *triangles* for leaves from BP and *squares* for leaves from GTR). Results of statistical analyses given in Table 3. For other abbreviations, see Fig. 1

At MAB, concentrations of FAAs and amines combined, Asn and Gln combined and Pro increased significantly between October and November (Fig. 4a, c, d; Table 3) but the proportional contribution of essential FAAs did not (Fig. 4b; Table 3). Interestingly, these same trends were apparent where there were feeding nymphs (BP and GTR) but increases between months were more pronounced than at MAB (with the exception of essential FAAs) (Fig. 4; Table 3).

**Table 3:** Concentrations of free amino acids (*FAAs*) and amine metabolites in leaves of *E. moluccana* at MAB (almost no psyllids), BP (high abundance) and GTR (very high abundance)

	*df*	*F*	*P*	Homogeneous subsets of trees^a^		
				A	B	C
GLM results						
All FAAs + amines						
MAB: Month	1, 60	13.27	0.005			
MAB: Tree (Month)	10, 60	1.52	0.155			
BP: Month	1, 60	29.69	<0.001			
BP: Tree (Month)	10, 60	2.62	0.010			
GTR: Month	1, 60	24.75	0.001			
GTR: Tree (Month)	10, 60	2.55	0.012			
% Essential FAAs						
MAB: Month	1, 60	0.53	0.483			
MAB: Tree (Month)	10, 60	5.33	<0.001			
BP: Month	1, 60	2.55	0.141			
BP: Tree (Month)	10, 60	3.43	0.001			
GTR: Month	1, 60	3.59	0.087			
GTR: Tree (Month)	10, 60	4.91	<0.001			
Asn + Gln						
MAB: Month	1, 60	8.59	0.015			
MAB: Tree (Month)	10, 60	1.45	0.181			
BP: Month	1, 60	39.50	<0.001			
BP: Tree (Month)	10, 60	2.61	0.011			
GTR: Month	1, 60	15.53	0.003			
GTR: Tree (Month)	10, 60	2.43	0.017			
Pro						
MAB: Month	1, 60	148.25	<0.001			
MAB: Tree (Month)	10, 60	0.88	0.560			
BP: Month	1, 60	12.38	0.006			
BP: Tree (Month)	10, 60	5.80	<0.001			
GTR: Month	1, 60	8.13	0.017			
GTR: Tree (Month)	10, 60	5.64	<0.001			
Repeated-measures GLM results						
All FAAs + amines						
MAB: Tree	5, 30	1.59	0.193	All trees (2.88)		
BP: Tree	5, 30	3.36	0.016	6 (4.87), 3, 4, 5, 2	1 (7.22)	
GTR: Tree	5, 30	2.04	0.102	All trees (8.98)		
% Essential FAAs						
MAB: Tree	5, 30	7.62	<0.001	4 (9.5), 6, 2, 3, 1	5 (19.5)	
BP: Tree	5, 30	4.28	0.005	4 (7.1), 2, 6, 3, 5	1 (13.0)	
GTR: Tree	5, 30	6.94	<0.001	1 (9.0), 2, 4, 5	6, 3 (18.1)	
Asn + Gln						
MAB: Tree	5, 30	1.13	0.366	All trees (161.7)		
BP: Tree	5, 30	3.63	0.011	6 (294.3), 3, 5, 4	1, 2 (1,031.5)	
GTR: Tree	5, 30	2.19	0.081	All trees (1,069.9)		
Pro						
MAB: Tree	5, 30	1.47	0.229	All trees (67.0)		
BP: Tree	5, 30	10.40	<0.001	4 (77.3), 6, 3, 5, 2	1 (381.5)	
GTR: Tree	5, 30	4.75	0.003	3 (143.4)	6, 2, 5, 4	1 (753.8)

*Asn* Asparagine,* Gln* glutamine, *Pro* proline; for other abbreviations, see Table 1

^a^ *Numbers in parentheses* are per tree average concentrations (all FAAs + amines in nmol mg^−1^ and other concentrations in pmol mg^−1^) or percentages (essential FAAs); *numbers within a row* identify individual trees at each site

For the most part, GLMs and repeated-measures GLMs indicated there were statistically significant between-tree differences in concentrations of FAAs (Table 3). The exceptions to this trend were for trees at MAB where concentrations of FAAs and amines combined, Asn + Gln and Pro were comparable. The percentage essential FAAs in leaves of most trees at MAB was comparable with the exception of concentrations for tree 5, which were almost double those in leaves of tree 3, which had the lowest concentration of essential FAAs (Table 3). At BP, tree 1 had the highest or equal highest concentrations of FAAs and amines and representation of essential FAAs; tree 1 had comparably high concentrations of Asn + Gln to tree 2. Only the percentage of essential FAAs and the concentration of Pro in leaves of trees at GTR exhibited statistically significant between-tree differences (Table 3). At GTR, the representation of essential FAAs was greatest in leaves from trees 3 and 6 and leaves of tree 1 had the highest concentrations of Pro.

### Size of nymphs

Differences in morphometric measurements related to site were most pronounced in fourth and fifth instar nymphs and minimal in first and second instar nymphs. MANOVA results for first and second instar nymphs were *F*_2,130_ = 3.845, *P* = 0.024 and *F*_2,104_ = 0.384, *P* = 0.682, respectively. One-way ANOVAs for first instar nymphs revealed that HW measurements for specimens from GTR were significantly larger than those for specimens from BP (Table 4).

**Table 4:** Mean (±SD) size of *Cardiaspina* near *densitexta* nymphs from BP (high abundance) and GTR (very high abundance) and results of one-way ANOVAs

	BP	GTR	*df*	*F*	*P*
I instar	*n* = 99	*n* = 34			
HW	0.203 ± 0.015	0.209 ± 0.013	1, 131	4.77	0.031
BL	0.383 ± 0.037	0.383 ± 0.027	1, 131	0.00	0.980
II instar	*n* = 19	*n* = 88			
HW	0.299 ± 0.053	0.293 ± 0.026	1, 105	0.60	0.441
BL	0.530 ± 0.118	0.523 ± 0.060	1, 105	0.14	0.706
IV instar	*n* = 42	*n* = 71			
HW	0.586 ± 0.059	0.531 ± 0.062	1, 111	21.23	<0.001
BL	1.112 ± 0.180	0.979 ± 0.185	1, 111	13.80	<0.001
FWL	0.262 ± 0.019	0.254 ± 0.021	1, 111	4.20	0.043
V instar	*n* = 90	*n* = 63			
HW	0.722 ± 0.052	0.699 ± 0.059	1, 151	6.30	0.013
BL	1.720 ± 0.332	1.672 ± 0.244	1, 151	0.96	0.328
FWL	0.692 ± 0.080	0.662 ± 0.072	1, 151	5.59	0.019
V instar ♂	*n* = 43	*n* = 35			
HW	0.689 ± 0.046	0.670 ± 0.056	1, 76	2.75	0.101
BL	1.636 ± 0.278	1.643 ± 0.267	1, 76	0.01	0.909
FWL	0.616 ± 0.026	0.608 ± 0.042	1, 76	1.08	0.302
V instar ♀	*n* = 47	*n* = 28			
HW	0.752 ± 0.037	0.735 ± 0.041	1, 73	3.10	0.082
BL	1.797 ± 0.360	1.708 ± 0.210	1, 73	1.42	0.237
FWL	0.762 ± 0.036	0.730 ± 0.035	1, 73	13.83	<0.001

*HW* Head width,* BL* body length,* FWL* forewing pad length; for other abbreviations, see Table 1

Morphometric measurements for fourth instar nymphs differed according to site (MANOVA, *F*_3,109_ = 7.061, *P* < 0.001). One-way ANOVAs indicated that HW and BL measurements of fourth instar nymphs from BP were significantly larger than those of nymphs from GTR (Table 4). Fourth instar nymphs from BP also had larger FWL measurements but differences were less pronounced. The MANOVA for all fifth instar nymphs (males and females combined) suggested that differences in morphological measurements between sites were minimal (*F*_3,149_ = 2.561, *P* = 0.057). One-way ANOVAs indicated that HW and FWL measurements for fifth instar nymphs from BP were larger than those for nymphs from GTR (Table 4). Partitioning measurements of fifth instar nymphs into those from probable males and probable females resulted in two distinct MANOVA results; the MANOVA for males indicated that measurements did not differ between sites (*F*_3,74_ = 2.398, *P* = 0.075) whereas the MANOVA for females indicated that measurements differed between sites (*F*_3,71_ = 4.585, *P* = 0.005). One-way ANOVAs for each of the three morphometric measurements from male fifth instar nymphs were non-significant (Table 4). In contrast, FWL measurements for female fifth instar nymphs from BP were significantly larger than those for nymphs from GTR.

### Psyllid-host interactions

Linear regression confirmed that the abundance of feeding nymphs on a leaf was negatively correlated with reduced total chlorophyll concentration (Table 5); this model explained 30.2 % of the variation.

**Table 5:** Models of effects of feeding by *Cardiaspina* near *densitexta* nymphs on *E. moluccana*

	PC	Adjusted *r* ^2^ (%)	Std slope	*df*	*F*-ratio	*P*
Foliar pigments						
Total chlorophyll	1	30.2	−0.553	1, 207	91.10	<0.001
FAAs + amines						
Ile, Leu, Lys, Thr, Trp, Val, Arg, Pro	1	27.1	0.524	1, 214	80.79	<0.001
Asp, Glu	2	33.8	0.265	2, 213	55.98	<0.001
Ala, GABA	3	35.2	0.130	3, 212	39.98	<0.001

*PC* Principal component, *Ile* isoleucine,* Leu* leucine, *Lys* lysine,* Thr* threonine,* Trp* tryptophan,* Val* valine, *Asp* aspartic acid, *Arg* arginine,* GABA* γ-aminobutyric acid

Step-wise regressions (based on groupings of variables identified by factor analysis) confirmed that the abundance of feeding nymphs was positively correlated with foliar concentrations of FAAs and amine metabolites (Table 5). The first factor, which explained 27.1 % of variation, comprised six essential amino acids [isoleucine (Ile), leucine (Leu), lysine (Lys), threonine (Thr), tryptophan (Trp) and valine (Val)] and two non-essential amino acids (arginine and Pro). The percentage of variation explained reached a maximum of approximately 35 % by inclusion in the regression of the third component factor.

## Discussion

*Cardiaspina* psyllid species are mature (expanded) leaf specialists of eucalypts. Consequently, *Cardiaspina* nymphs must derive nutrition from modules which have declined in quality since they were first initiated. Moreover, because undisturbed nymphs are sessile they are only able to access host tissues in the region beneath their lerp. Both these facts may have exerted strong selective pressure on species within the genus to enhance the nutritional quality of modules to facilitate nymphal survival and performance. This study has shown that feeding by *Cardiaspina* nymphs is associated with the chlorosis of host leaves which increases in extent and severity as nymphs grow. Chlorosis was also associated with increased concentrations of FAAs and amine metabolites but only very high numbers of feeding nymphs were associated with significantly elevated concentrations of essential amino acids. Importantly, in the absence of intra-specific competition (e.g. during irruptions ), elevated foliar amino N was associated with improved nymphal performance (indicated by body size). The processes initiated by feeding nymphs bear many similarities to those known to occur during natural leaf senescence which could be interpreted from either an insect manipulation or plant defence perspective.

The term ‘nutritional enhancement’ was originally proposed to describe the effects that feeding by some species of aphid have on their host plants (Sandström et al. 2000). We can now confirm White’s (1970) suggestion that *Cardiaspina* nymphs also nutritionally enhance eucalypt host tissues. Taylor (1997) showed that discolouration of *Eucalyptus camaldulensis* leaves induced by feeding *Cardiaspina albitextura* Taylor nymphs was associated with an increase in total free amino N and in total phenol concentrations. These changes coincided with the collapse of *C. albitextura* populations. To our knowledge, the only other study to apparently demonstrate that feeding by psyllid nymphs induces nutritional enhancement of host leaves is that by Yang et al. (2011). These authors found that feeding by adults (for only 7 days) of a triozid species of psyllid, *Bactericera cockerelli* (Šulc), was associated with increased foliar concentrations of some FAAs (Leu, serine and Pro) and decreased concentrations of others (Glu, aspartic acid and Lys). Since the adult psyllids confined on potato leaves by these authors tested positive for the liberibacter *Candidatus Liberibacter psyllaurous* (synonym solanacearum) (responsible for Zebra chip disease), and changes in FAAs following feeding by pathogen-free psyllids were not considered, it is impossible to attribute the changes measured to either the pathogen or the psyllid. To date, no eucalypt-feeding psyllid has been reported to vector a plant pathogen harmful to its host. Moreover, because leaf discolouration by feeding *C. densitexta* nymphs requires at least 1 week to appear and eventual necrosis of affected areas is delayed until after a feeding site is vacated (White 1970), it seems unlikely that a self-replicating agent is responsible for the changes in FAAs we measured. White (1970) concluded that a salivary toxin was responsible for the discolouration of *E. fasciculosa* leaves by *C. densitexta* nymphs.

If nutritional enhancement were beneficial to nymphs, there should be evidence of enhanced survival and/or performance (H3). Unexpectedly, fourth instar nymphs from leaves with fewer feeding nymphs (BP) were larger than those from leaves with greater numbers of nymphs (GTR). This trend was still apparent by the fifth instar but was attributed to differences in the morphometric measurements of female nymphs only and pertained specifically to differences in their FWL measurements. We attribute the smaller size of nymphs from GTR compared to those from BP to intra-specific competition for FAAs in leaves of hosts at GTR. Hence, if nymphs accrue any nutritional benefit from the mobilisation of amino N, it seems most likely to occur at low to moderate nymphal densities per leaf rather than at very high (irruption) densities. Since female psyllids can be much larger than males (i.e. up to 1.3 times larger), our evidence suggests that nutritional enhancement could benefit female vagility (and hence dispersal capacity) and fecundity. A number of authors have shown that psyllid body size/weight is responsive to the nutritional quality of their hosts, although none have linked psyllid performance to specific FAAs (Webb and Moran 1978; Sutton 1984; Thomson et al. 2001). Whilst feeding by adult *C. densitexta* is not associated with any apparent phytotoxic response (White 1970), possibly because they do not remain feeding in the one location for long periods as do nymphs, the senior author has observed aggregations of adults feeding from chlorotic and reddish areas associated with occupied and abandoned lerps possibly suggesting that adults prefer these areas to healthy tissues. Hence, adult *Cardiaspina* may detect the changes in leaf colour associated with nymph-induced senescence and, by virtue of their greater mobility, be able to exploit their heightened nutritional quality (see Holopainen et al. 2009).

We provide quantitative data on the extent to which feeding nymphs affect the concentrations of three photosynthetic pigments. At GTR, feeding nymphs reduced total chlorophyll, carotenoids and anthocyanins concentrations by an average of 2.4, 1.8 and 1.7 times, respectively. Comparable reductions in concentrations of foliar pigments were not observed where the densities of nymphs were markedly lower (BP). At BP, total chlorophyll exhibited the greatest between-tree variation in concentrations linked to the density of nymphs; most trees showed little difference in carotenoid and anthocyanin concentrations. Where there were virtually no feeding nymphs (MAB), we measured statistically significant between-tree differences in all three photosynthetic pigments. This indicates that there is inherent variation in the quality of trees which may influence psyllid host selection behaviours. We assume that the reductions in total chlorophyll at GTR must have been associated with reductions in the photosynthetic capacity of infested leaves because the losses were much greater than those not found to reduce the photosynthetic capacity of naturally senescing eucalypt leaves (Wendler et al. 1995). If this assumption is correct, it argues against an insect manipulation interpretation of host utilisation by this psyllid (H1). For example, inhabited galls of the triozid psyllid *Schedotrioza multitudinea* (Maskell) remain green if shaded or turn red when nymphs reach the third or later instars if exposed to sunlight, suggesting no adverse impact on the photosynthetic capacity of galled leaf tissues by their manipulation of the host’s physiology (Taylor 1985). That the intensity of foliar chlorosis was positively correlated with the abundance of nymphs is evidence supportive of a plant defence interpretation of responses to psyllid feeding (H4).

The findings of Crawford and Wilkens (1996) explain the elevated concentrations of FAAs we measured through the lysis of thylakoid membranes within chloroplasts. Destruction of the thylakoid membranes would liberate chlorophyll molecules, permitting them to be catabolised and ingested by nymphs; the result of either fate is foliar chlorosis. Given this scenario, the FAAs liberated by the salivary enzymes of psyllid nymphs must reflect the composition of the major proteins forming the thylakoid membranes of the chloroplasts of *E. moluccana* leaves. Hence, it seems unlikely that *Cardiaspina* nymphs can selectively elevate concentrations of specific amino acids essential for their development—as would be predicted assuming an insect host manipulation interpretation (H2). Of the six essential amino acids (Ile, Leu, Lys, Thr, Trp and Val) found to be elevated in leaves of *E. moluccana* infested by nymphs, four were the same as those associated with higher abundances of a free-living, non-senescence-inducing psyllid species, *Ctenarytaina eucalypti* (Maskell), on *Eucalyptus globulus* Labill., i.e. Ile, Leu, Thr, Val and methionine (Met) (Steinbauer 2013). Met was identified as positively correlated with the abundance of *C. eucalypti* nymphs whereas Trp was positively correlated with the abundance of *Cardiaspina* nymphs. In the case of *C. eucalypti*, Lys was negatively correlated with the abundance of nymphs, which reflects that this species is a shoot-feeding specialist and that the young leaves it is associated with have lower concentrations of Lys compared to older leaves (Steinbauer 2013). Decreased foliar Ile concentrations have been implicated in the decline of *Strophingia ericae* (Curtis) populations on heather (Salt et al. 1998). Bioassays using artificial media are required to identify the essential amino acids linked to the survival and performance of *Cardiaspina* nymphs.

The regression results indicate that the abundance of nymphs only explained about one-third of changes in total chlorophyll and FAAs and amine metabolite concentrations. Consequently, factors other than nymphal feeding must have contributed to the plant responses we recorded. Data from MAB indicated that between-tree variation and natural leaf senescence contributed to some declines in foliar pigments and, to a lesser extent, some mobilisation of amino N. Assuming a background of between-tree variation in physiology and a basal rate of amino N mobilisation from natural leaf senescence, a physiological stress other than nymphal feeding not accounted for is photodamage. Pro concentrations were up to five times higher in some trees at BP and GTR but the highest concentration at BP (tree 1) was only half that of the highest concentration at GTR (tree 1). Leaves at BP and especially at GTR may have experienced oxidative pressure as a consequence of locally reduced photosynthetic capacity and depleted tissue (non-amino) N leading to photoinhibition (Close and McArthur 2002; Close and Beadle 2003). In addition, concentrations of carotenoids (which possess antioxidant activity) and anthocyanins at GTR were decreased substantially by feeding by late-instar nymphs which may have further exacerbated the oxidative pressure on leaves at this site. Highly reactive chemical species, the cause of photoinhibition, could cause further damage to thylakoid membranes and liberate FAAs. Nevertheless, the visible symptom of severe photoinhibition, the synthesis of high concentrations of anthocyanins, was not observed, presumably because morning temperatures were warm to high. Close et al. (2000) reported that cold and sunny conditions after frost (e.g. in the morning) were associated with increased photoinhibition. As mentioned, anthocyanins act as light (visible) screening pigments providing photoprotection and have generally not been shown to have harmful effects on insect herbivores (Close and Beadle 2003; Rostás et al. 2002; Ramírez et al. 2008). Although it may be unlikely that anthocyanins have antibiotic activity against psyllids, increases in their concentration are positively linked to increased phenol synthesis (Close et al. 2003; Karageorgou and Manetas 2006). Consequently, concomitant high concentrations of other phenolic compounds (e.g. quercetin) could adversely affect psyllid survival but actual effects remain to be quantified. Morgan and Taylor (1988) made reference to the possible antibiotic effect of quercetin on *Cardiaspina* nymphs because Farr (1985) reported that it decreased N assimilation by larvae of the gum leaf skeletoniser, *Uraba lugens* Walker, more than other phenols assayed (namely caffeic, chlorogenic and gallic acids). Again, bioassays will be necessary to quantify effects of phenolic compounds on the survival of *Cardiaspina* nymphs.

Necrosis of psyllid-induced chlorotic lesions was not observed when nymphs were either still feeding (October) or most had just eclosed (November; presumably when salivary enzyme titres had only just begun to decline). Necrosis of anthocyanic lesions follows eclosion of *Cardiaspina* nymphs (White 1970; Morgan and Taylor 1988). This suggests that feeding by nymphs prevents the onset of severe water stress of mesophyll cells which is associated with natural leaf senescence (Crawford and Wilkens 1996). The prevention of the dehydration and necrosis of feeding lesions is the plant physiological response that nymphs must manipulate since these states would be lethal to them. Since tissue necrosis occurs after nymphs have eclosed, it cannot be considered a plant defence. Furthermore, because anthocyanin synthesis precedes necrosis, it is consistent with natural leaf senescence. Although Pegadaraju et al. (2005) state that aphid feeding induced premature chlorosis and cell death (i.e. symptoms of hypersenescence), they present no evidence concerning the fate of individual aphids on such plants. They reported fewer aphids from a hypersenescent mutant (i.e. pad4-1) compared to a wild type but provide no explanation (nutritional or physical) for the differences in abundance they recorded. Significantly, White (1970) found that leaf discs floated on distilled water containing aqueous extracts of ground nymphs turned necrotic much faster than those maintained on just distilled water. Perhaps this factor is released into chlorotic lesions when nymphs withdraw their stylets prior to eclosion.

Feeding by *Cardiaspina* nymphs causes chlorosis and is not apparently associated with the de novo synthesis of unique nutrients, both of which do not fit an insect manipulation interpretation. It is probable that nutritional enhancement benefits the performance of nymphs in the absence of intra-specific competition and feeding also delays the onset of leaf necrosis—both of which are consistent with a manipulation interpretation. Although we did not quantify changes in potentially harmful secondary metabolites, feeding induces few plant responses that can be readily equated with a plant defence interpretation. On balance, nutritional enhancement of leaves by *Cardiaspina* nymphs through induction of premature senescence fits better with a plant-wounding interpretation. Better resolution of the processes initiated in the leaves of host eucalypts by feeding *Cardiaspina* nymphs will require identification of the salivary enzymes that psyllids secrete into plant tissues and investigation of the biosynthetic pathways affected by these enzymes.
